# Similar brain proteomic signatures in Alzheimer’s disease and epilepsy

**DOI:** 10.1002/alz.091313

**Published:** 2025-01-03

**Authors:** Dominique Leitner, Geoffrey Pires, Tomas Kavanagh, Evgeny Kanshin, Manor Askenazi, Beatrix Ueberheide, Orrin Devinsky, Eleanor Drummond, Thomas Wisniewski

**Affiliations:** ^1^ NYU Grossman School of Medicine, New York, NY USA; ^2^ The University of Sydney, Sydney, NSW Australia; ^3^ Biomedical Hosting LLC, Arlington, MA USA

## Abstract

**Background:**

Prevalence of epilepsy is increased among Alzheimer’s Disease (AD) patients and cognitive impairment is common among people with epilepsy, with seizures occurring in 10‐22% of AD patients, subclinical epileptiform abnormalities in 22‐54% of AD patients, and cognitive deficits in up to 80% of epilepsy patients. These shared pathophysiological changes remain poorly defined.

**Methods:**

We aimed to identify protein differences associated with epilepsy and AD using published proteomics datasets, including our previous analysis in epilepsy hippocampus and from our searchable database of 38 AD proteomics studies (NeuroPro). Additionally, laser capture microdissection was performed in dual diagnosed (AD+epilepsy) cases, epilepsy, AD, and control cases in hippocampal CA1‐3 (n = 7/group), followed by label‐free quantitative mass spectrometry to identify protein differences.

**Results:**

We observed a significant overlap in protein differences in epilepsy and AD: 89% (689/777) of proteins altered in the hippocampus of epilepsy patients were significantly altered in advanced AD. Of the proteins altered in both epilepsy and AD, 340 were altered in the same direction, while 216 proteins were altered in the opposite direction. Synapse and mitochondrial proteins were markedly decreased in epilepsy and AD, suggesting common disease mechanisms. In contrast, ribosome proteins were increased in epilepsy but decreased in AD. Notably, many of the proteins altered in epilepsy interact with tau or are regulated by tau. This suggests tau likely mediates common protein changes in epilepsy and AD. Immunohistochemistry for amyloid‐beta and multiple phosphorylated tau species (pTau396/404, pTau217, pTau231) showed a trend for increased intraneuronal pTau217 and pTau231 but no phosphorylated tau aggregates or amyloid plaques in epilepsy hippocampal sections. To better understand mechanisms of epilepsy in AD, we performed localized proteomics in the hippocampal CA1‐3 of dual diagnosed cases. We observed protein changes compared to controls (275 proteins), with fewer differences than either AD (67) or epilepsy (87) alone cases. Altered proteins in Dual vs. AD included those associated with mTOR signalling related pathways, regulation of eIF4 and p70S6K (p = 1.62 × 10^‐4^) and mTOR signalling (p = 2.19 × 10^‐2^).

**Conclusion:**

Our results provide insights into common mechanisms in epilepsy and AD and highlight the potential role of tau and mTOR signalling in both disease groups.

